# Measurement of nicotine withdrawal symptoms: linguistic validation of the Wisconsin Smoking Withdrawal Scale (WSWS) in Malay

**DOI:** 10.1186/1471-2288-10-46

**Published:** 2010-05-22

**Authors:** Ahmed Awaisu, Sulastri Samsudin, Nur A Amir, Che G Omar, Mohd I Hashim, Mohamed H Nik Mohamad, Asrul A Shafie, Mohamed A Hassali

**Affiliations:** 1 Discipline of Clinical Pharmacy, School of Pharmaceutical Sciences, Universiti Sains Malaysia, 11800 Penang, Malaysia; 2 National Poison Centre, Universiti Sains Malaysia, 11800 Minden, Penang, Malaysia; 3 Advanced Medical and Dental Institute, Universiti Sains Malaysia, Taman Bertam Indah,13200 Kepala Batas, Penang, Malaysia; 4 Department of Pharmacy Practice, Kulliyyah of Pharmacy, International Islamic University Malaysia, Jln Sultan Ahmad Shah, 25200 Kuantan, Malaysia; 5 Discipline of Social and Administrative Pharmacy, School of Pharmaceutical Sciences, Universiti Sains Malaysia, 11800 Minden, Penang, Malaysia

## Abstract

**Background:**

The purpose of the linguistic validation of the Wisconsin Smoking Withdrawal Scale (WSWS) was to produce a translated version in Malay language which was "conceptually equivalent" to the original U.S. English version for use in clinical practice and research.

**Methods:**

A seven-member translation committee conducted the translation process using the following methodology: production of two independent forward translations; comparison and reconciliation of the translations; backward translation of the first reconciled version; comparison of the original WSWS and the backward version leading to the production of the second reconciled version; pilot testing and review of the translation, and finalization.

**Results:**

Linguistic and conceptual issues arose during the process of translating the instrument, particularly pertaining to the title, instructions, and some of the items of the scale. In addition, the researchers had to find culturally acceptable equivalents for some terms and idiomatic phrases. Notable among these include expressions such as "irritability", "feeling upbeat", and "nibbling on snacks", which had to be replaced by culturally acceptable expressions. During cognitive debriefing and clinician's review processes, the Malay translated version of WSWS was found to be easily comprehensible, clear, and appropriate for the smoking withdrawal symptoms intended to be measured.

**Conclusions:**

We applied a rigorous translation method to ensure conceptual equivalence and acceptability of WSWS in Malay prior to its utilization in research and clinical practice. However, to complete the cultural adaptation process, future psychometric validation is planned to be conducted among Malay speakers.

## Background

Tobacco smoking is now a global epidemic of public health concern. Current reports conclude that cigarette smoking is the largest avoidable cause of premature death and disability worldwide [1,2]. Tobacco now kills about 5 million people worldwide and if current trend continues, 10 million smokers per year are projected to die by 2025 [2,3]. In Malaysia, the prevalence of smoking is moderately high and the Third National Health and Morbidity Survey has reported an overall smoking rate of 21.5%, and rates of 46.4% and 1.6% among male and female above 18 years of age, respectively [4]. The mortality rate from smoking-related respiratory diseases in Malaysia was 1017 per 100,000 population [5]. The Malaysian government has initiated smoking cessation clinics as part of its National Tobacco Control Program (NTCP).

Cigarette and other forms of tobacco are addictive in nature due to the nicotine contained in them. Nicotine dependence causes physical withdrawal as well as lifelong addiction. When tobacco use is stopped, nicotine withdrawal syndrome emerges, because the body has developed a homoeostatic response [2,6,7]. The withdrawal syndrome is characterized by symptoms such as irritability, frustration or anger, restlessness, insomnia, anxiety, depression, problems getting along with friends and family, poor concentration, increased appetite, and craving for tobacco [2,7,8]. Unfortunately, this syndrome is an important impediment to successful quitting and may lead to smoking relapse. Therefore, an effective counselling for smoking cessation should emphasize coping skills with stress and withdrawal symptoms as well as provision of social support as part of the treatment [9].

Consequently, assessment of withdrawal symptoms form an integral part of assessing health and quality of life in smokers during the process of quitting smoking as well as an important determinant of success. Accurate and efficient measurement of withdrawal symptoms is important theoretically and clinically in helping to understand nicotine dependence and in developing better treatment methods [8,10]. A number of questionnaires with varying coverage of symptoms, quantitative indices of withdrawal and psychometric properties have been developed for this purpose [10,11]. The Wisconsin Smoking Withdrawal Scale (WSWS), originally developed in U.S. English [8], is one of such instruments used to measure tobacco withdrawal symptoms experienced during the process of quitting smoking. This patient-reported outcome measure combines the strength of sensitivity to smoking withdrawal and is predictive of smoking cessation outcomes. Furthermore, WSWS is sufficiently brief to be used in routine clinical practice and research context [8,10,12]. Prior to use in international trials, the measure underwent linguistic validation in 18 languages [13].

Despite high smoking prevalence, several ongoing clinical studies related to smoking cessation in Malaysia and the obvious advantages of using WSWS as an indicator of nicotine withdrawal symptoms, there were no translated versions of any type of scale for the assessment of nicotine withdrawal in Malay for use in clinical research and routine clinical practice in Malaysia or other Malay-speaking populations. We therefore subjected this WSWS scale to linguistic validation and cultural adaptation processes in Malaysia for future use by clinicians and researchers.

## Methods

The development of patient-reported outcomes measures such as WSWS for cross-cultural comparisons requires achieving "conceptual equivalence" between the original instrument and the target translation instrument [14-16]. Conceptual equivalence in the context of the current study is the absence of differences in meaning and content between the WSWS source language (U.S. English) and the translated version (Malay) [16]. This is achieved through a process called linguistic validation and cultural adaptation [14,15,17], as described henceforth. The cultural adaptation encompasses two essential and complementary stages: a translation stage to achieve linguistic validity of the questionnaire in the target language and psychometric evaluation. This paper aims to describe the linguistic validation of WSWS to produce a translated version in Malay language that was conceptually equivalent to the original version, as well as clear and easy to understand. Approval for the conduct of the study was obtained from the Human Research Ethics Committee of the Universiti Sains Malaysia.

### Professional requirements

A seven-member translation committee involving investigators from various schools and translators from School of Languages, Literacy and Translation (SoLLaT) at Universiti Sains Malaysia was formed. The professional translators from SoLLaT were selected based on their cognate experiences and track record of success in linguistic validation of quality of life or similar instruments. The WSWS validation was conducted in close collaboration between the translation committee comprising the above individuals and the copyright owner/developers of the questionnaire. The linguistic validation processes outlined in the methodology section comprise of the general procedures derived from the literature and internationally accepted methodology [13-18].

### Description and psychometric properties of the original instrument

The 28-item WSWS contains seven reliable subscales, tapping the major symptom elements of the nicotine withdrawal syndromes [8]. The seven subscales include: anger, anxiety, sadness, concentration, hunger, sleep, and craving. These domains contain DSM symptoms of nicotine withdrawal [7]. The reliability measures for all the subscales using Cronbach's α were appreciable (coefficients α for the subscales range from 0.75 to 0.93). Moreover, the questionnaire is sensitive to smoking withdrawal and is predictive of smoking cessation outcomes. As other questionnaires, the scale is composed of instructions, items and corresponding response choices. The items are scored on a 5-point Likert type scale (0 = strongly disagree, 4 = strongly agree).

### The linguistic validation processes

**Figure 1:**
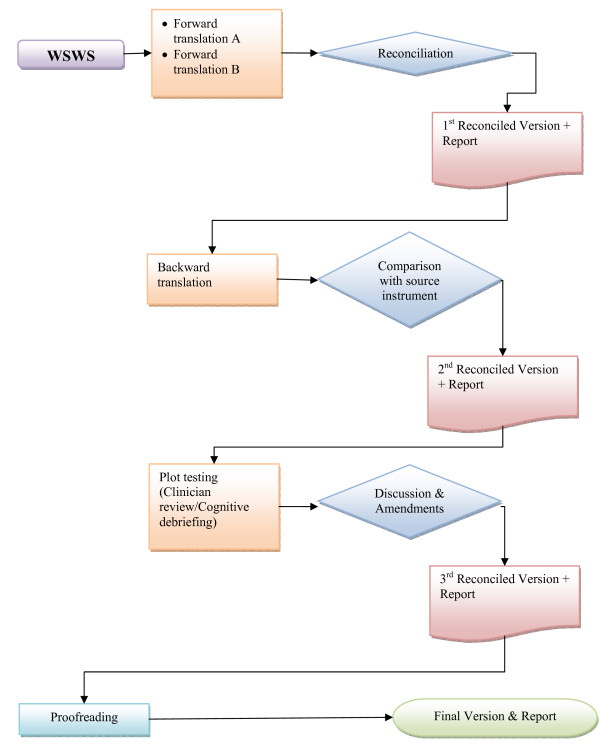
**Algorithm of the linguistic validation processes**.

The linguistic validation of the WSWS consists of three major steps: forward translation, backward translation for quality control, and pilot testing. Each stage helps to improve the quality of the translation in terms of conceptual equivalence and ease of understanding of potential users. A schematic presentation of the entire process used in this study is illustrated in Figure 1.

#### Forward translation

We obtained permission for the cultural adaptation and linguistic validation of the WSWS in Malay from the original questionnaire developers and the copyright owner at the University of Wisconsin Center for Tobacco Research and Intervention. All the concepts in the original questionnaire were clearly defined. The aim of the forward translation step was to produce a version in the target language (Malay) that is conceptually equivalent and close to the original WSWS in meaning. Two professional translators, who are native Malay speakers, bilingual and proficient in English, undertook independent forward translations of the original English questionnaire into Malay [14,15,17]. Comparison and reconciliation of the translations was done in a series of meetings between the investigators and the translators. Linguistic issues that arose were resolved by way of further consultations and consensus. The first reconciled version was developed in Malay based on the two forward translations and reconciliation.

#### Backward translation

A backward translation of the reconciled language version was produced in the source language (U.S. English) by a professional translator, native English speaker and bilingual in the Malay language [14,15,17]. The backward translation was compared to the original WSWS, leading to the production of the second reconciled version in Malay. Similarly, during this process, translation discrepancies and linguistic issues arose, and modifications were made accordingly.

#### Pilot testing (clinician's review and cognitive debriefing)

Two clinicians with cognate experience in smoking cessation reviewed the second Malay reconciled version of WSWS. This step aimed to obtain feedback from experts in the relevant medical field. Relevant feedbacks were incorporated into the second reconciled Malay version. A cognitive debriefing using face-to-face interview was further conducted. The translated questionnaire (second reconciled version) was administered on a sample of seven smokers with low to high level of education who were at different stages of quitting smoking [15,17]. The sample was conveniently selected based on a sampling frame available at a quit smoking clinic at Universiti Sains Malaysia. The interview was conducted by investigators from the translation team using both rephrasing and probing techniques. The aim of this step was to ensure that the translation (instructions, items and response choices) was acceptable, understood in the way it was intended to be, and the language used was simple and appropriate for the target future users of the scale. The third reconciled Malay version was produced based on the results of the clinicians' review and respondents' feedback. A summary report was compiled at each stage of the process.

#### Finalization

Finally, the third reconciled version was proofread by a native Malay speaker in order to ensure that the final version was devoid of typographical, spelling and grammatical errors. Consequently, the final version of WSWS questionnaire in Malay was produced for clinical use (see Additional File 1: Appendix).

## Results

Here we present a summary of the linguistic issues and problems encountered during the linguistic validation processes and the necessary measures taken to resolve them. Grammatical and spelling errors corrected during proofreading of the third reconciled version of the translated questionnaire are not presented here.

### Title of the instrument: "Wisconsin Smoking Withdrawal Scale (WSWS)"

The title of the source instrument was "Wisconsin Smoking Withdrawal Scale (WSWS)". In Malay language, the term "withdrawal" literally translates to "*penarikan*" ("to pull something"). The term withdrawal which is more of a technical term is not easily understandable in spoken Malay language. Therefore, the literal translation of the instrument's title was not possible in Malay; thus, the whole title was rendered by a culturally acceptable linguistic equivalent. The translation committee agreed to use the phrase '*gejala penarikan*' instead of '*penarikan*', which means 'withdrawal symptoms'. The term '*selepas berhenti merokok*' was further added to make the term withdrawal more meaningful and understandable for the Malay respondents. Consequently, the title was rendered to "***Skala Wisconsin Untuk Gejala Penarikan Selepas Berhenti Merokok" ***(Wisconsin Scale for Withdrawal Symptoms after Quitting Smoking), which was more acceptable in Malay.

### Instructions

The first instruction sentence: "Please answer the following questions based on how you have felt or what you have noticed [over the last 24 hours/over the last week]".

The second instruction sentence: "Answer based on how you have felt in general during this time".

The second sentence of the instructions was completely deleted, because translation of this sentence tended to make users confused especially in Malay. Users would generally assume that the item questions about their feelings "at the present moment", rather than over the stated period, as emphasized in the sentence preceding that (i.e. over the last 24 hours/over the last week). Furthermore, during the cognitive testing, respondents were generally confused about two phrases used in the first sentence of the instructions which were "*bagaimana perasaan anda*" (how you have felt) and "*apa yang anda sedari*" (what you have noticed). They were not sure whether these phrases were referring to their feelings during the process of quitting smoking or in natural behaviors. Owing to the persistent misconceptions and concerns raised by the cognitive debriefing subjects regarding the two phrases in the first sentence, we introduced a new sentence "*Jawapan anda mestilah berdasarkan pengalaman anda berhenti merokok"*, which translates to "Your answer should be based on your quitting experience". Therefore, the instructions were rendered to *Sila jawab soalan-soalan berikut berdasarkan "bagaimana perasaan anda" atau "apa yang anda sedari" (dalam tempoh 24 jam yang lalu/dalam tempoh seminggu yang lepas). Jawapan anda mestilah berdasarkan pengalaman anda berhenti merokok*, which carry the meaning Please answer the following questions based on "how you have felt" or "what you have noticed" (over the last 24 hours/over the last week). Your answer should be based on your quitting experience. These changes were made after pertinent consultations with the developers of the original instrument.

### Response choices

No issues arose or problems encountered regarding the response choices throughout the linguistic validation process.

### Idiomatic expressions and terms within the items

Culturally acceptable equivalents had to be found for the following terms and expressions within the items:

▪ irritability → easily angered (*mudah marah*)

▪ nibbling on snacks or sweets → urge to snack or have sweets (*hendak mengunyah snek atau gula-gula*)

▪ upbeat → joy (*gembira*)

Furthermore, the literal translation of some expressions within the items was not possible in Malay and they had to be rendered by culturally acceptable linguistic equivalents.

▪ Item 1: Food is not particularly appealing to me → I have no interest in food. (*Makanan bukanlah sesuatu yang menarik minat saya*)

▪ Item 14: I want to nibble on snacks or sweets → I feel the urge to snack or have sweets. (*Saya terasa hendak mengunyah snek atau gula-gula*)

▪ Item 26: I have trouble getting cigarettes off my mind → It is difficult for me to forget about cigarettes. (*Saya mempunyai masalah untuk melupakan rokok*)

During the pilot testing, the Malaysian Malay speakers more readily understand the meaning of the above expressions when they are rendered as the phrases following the arrows.

### Cognitive debriefing

The Malay translated second reconciled version of WSWS was tested on seven Malay respondents with a mean age of 29.6 years, who were in the process of quitting smoking or had prior experience of quitting. Respondents took an average of about 14 minutes to complete the questionnaire.

In general, the respondents did not encounter problems with understanding the contents of the Malay version of the WSWS. The instrument was found to be easily comprehensible, clear and appropriate for the smoking withdrawal symptoms intended to be measured. However, the subjects raised a concern on the first sentence of the instructions, which created some misconceptions. They provided some suggestions, a consensus was reached and we retained the item by providing a supplementary explanatory sentence. This issue has been previously discussed under the "instructions".

Furthermore, three of seven subjects involved in the cognitive debriefing raised a concern that Item 20: "I have thought about smoking a lot", sounds not specific to them (whether it was referring to smoking and health, frequency of smoking or the urge to smoke). Based on the discussions during the cognitive debriefing, the committee agreed to modify the item from "*Saya sering terfikir tentang merokok sejak akhir - akhir ini" *to *"Saya sering terfikir untuk merokok sejak akhir-akhir ini" *which means "I think about smoking a lot". The word "*tentang*" was replaced with "*untuk*" to strengthen the meaning of the sentence, which was meant to ask about the respondent's urge to smoke cigarettes. The subjects were satisfied with the questionnaire and provided favorable feedbacks. The cognitive debriefing process was therefore successful.

## Discussion

Smoking harms nearly every organ in the body, causing a wide range of diseases and reducing quality of life and life expectancy [1]. Moreover, withdrawal symptoms can be unpleasant and may cause smokers trying to quit to relapse. Therefore, measuring withdrawal symptoms and quality of life is a vital part of assessing health in smokers during the action or maintenance stage of quitting [8,13]. Cultural adaptation of quality of life instruments using standard procedures is becoming increasingly important in different countries and across different cultures. This is to ensure the optimal transfer of the original message and measuring what is intended to be measured. In the present study, linguistic validation of WSWS in Malay was carried out within the confines of internationally accepted guidelines [13-16,18]. Most importantly, the translated instrument should be understood by most respondents in the Malaysian population and should maintain a reading and comprehension level that will be accessible by most respondents, even of a low education level.

It should also be kept in mind that the questionnaire should always be considered as a whole (e.g., the wording of the response choices may influence the translation of the items, and vice-versa). During the translation process of WSWS in Malay, a considerable number of linguistic and semantic issues arose. Most notable were the title, instructions, and items 1, 14, 20, and 26 of the questionnaire. The forward and backward translation processes permitted changes to be made with respect to the U.S. English version where words or concepts were untranslatable or where words or terms have a specific meaning in English but a semantically different or secondary meaning in Malay. For instance, in Malay language, the term "withdrawal" literally translates to "*penarikan*" ("to pull something"). Using the term as such would be meaningless and not easily understandable in spoken Malay. The phrase '*gejala penarikan*' instead of '*penarikan*', which means 'withdrawal symptoms' had to be utilized. Moreover, in Malay language, there is no literal translation to the expression "trouble getting cigarettes off my mind" (see item 26) and this had to be rendered as "difficult for me to forget about cigarettes". The backward translator was actively involved in the production of the second reconciled version in order to detect any misunderstandings, mistranslations or inaccuracies in the intermediary forward version of the questionnaire. This might have reduced cultural and social bias that may result when only one or two translators are responsible for the translation. Overall, the actions taken in the resolution of the problems encountered during these processes involved consultations and collaborations with experts in linguistics and the original developers. This reinforces the credibility of a rigorous translation method to ensure conceptual equivalence [15,16]. Further, the cognitive interviewing techniques used can minimize sources of measurement error introduced by the translation process by detecting question items, terms, or response options that were difficult to understand or that were misunderstood by the respondents.

The availability of Malay version of WSWS has a promising role in helping clinicians and researchers providing smoking cessation interventions to better understand the withdrawal symptoms experienced by their patients and to individualize the treatments for tobacco dependence. In the light of the negative consequences associated with nicotine withdrawal syndrome on achieving abstinence, the authors recommend that all clinicians should use this tool as part of routine clinical practice in Malaysia.

## Conclusion

A rigorous translation method was applied to ensure conceptual equivalence and acceptability of WSWS in Malaysian Malay language prior to its utilization in smoking cessation studies or clinical practice. However, further psychometric testing should be conducted to ensure the validity and reliability of the translation and its appropriateness among the Malaysian population.

## Competing interests

The authors declare that they have no competing interests.

## Authors' contributions

AA and MHNM conceived the study, participated in the design of the study, coordinated the research and drafted the manuscript. SS, NAA, CGO, and MIH participated in the instrument validation processes and manuscript preparation. AAS and MAAH participated in the design of the study and instrument validation process, and helped in drafting the manuscript. All authors read and approved the final manuscript.

## Pre-publication history

The pre-publication history for this paper can be accessed here:

http://www.biomedcentral.com/1471-2288/10/46/prepub

## Supplementary Material

Additional file 1 **Appendix 1**. Malay Wisconsin Smoking Withdrawal Scale (WSWS). Click here for file
