# Supplementary material for: Measurement of nicotine withdrawal symptoms: linguistic validation of the Wisconsin Smoking Withdrawal Scale (WSWS) in Malay
Source: BMC Med Res Methodol. 2010 May 22;10:46. doi: 10.1186/1471-2288-10-46 (PMC2882374; doi:10.1186/1471-2288-10-46)
Supplement: Additional file 1 — Appendix 1. Malay Wisconsin Smoking Withdrawal Scale (WSWS). [file 1471-2288-10-46-S1.PDF]

Appendix I: Malay Wisconsin Smoking Withdrawal Scale (WSWS)

SKALA WISCONSIN UNTUK GEJALA PENARIKAN SELEPAS BERHENTI MEROKOK

Sila jawab soalan-soalan berikut berdasarkan ”bagaimana perasaan anda” atau ”apa yang anda sedari”. (dalam tempoh 24 jam yang lalu/dalam tempoh seminggu yang lepas). Jawapan anda mestilah berdasarkan pengalaman anda berhenti merokok.

0	1	2	3	4
Sangat tidak setuju	Tidak setuju	Rasa biasa	Setuju	Sangat setuju

Bil.	Perkara	Skor				
		Sangat tidak setuju	Tidak setuju	Rasa biasa	Setuju	Sangat setuju
1.*	Makanan bukanlah sesuatu yang menarik minat saya	0	1	2	3	4
2.*	Saya dapat tidur dengan nyenyak	0	1	2	3	4
3.	Saya rasa tertekan atau gelisah	0	1	2	3	4
4.*	Saya dapat memberi tumpuan terhadap sesuatu perkara dengan baik	0	1	2	3	4
5.	Saya sering terjaga daripada tidur pada waktu malam	0	1	2	3	4
6.	Saya mudah hilang sabar sejak akhir-akhir ini	0	1	2	3	4
7.*	Saya rasa gembira dan positif	0	1	2	3	4
8.	Saya bimbang tentang masalah diri saya sejak akhir-akhir ini	0	1	2	3	4
9.	Saya sering terasa desakan untuk merokok	0	1	2	3	4
10.*	Saya rasa tenang akhir - akhir ini	0	1	2	3	4
11.	Saya rasa terganggu dengan keinginan untuk merokok	0	1	2	3	4
12.	Saya rasa sedih atau murung	0	1	2	3	4
13.	Saya mudah marah sejak akhir-akhir ini	0	1	2	3	4
14.	Saya rasa hendak mengunyah snek atau gula-gula	0	1	2	3	4
15.	Saya rasa terganggu dengan sikap negatif (seperti berang, kecewa dan mudah marah) yang ada pada diri saya.	0	1	2	3	4
16.	Saya makan banyak sejak akhir-akhir ini	0	1	2	3	4
17.*	Saya berpuas hati dengan tidur saya	0	1	2	3	4
18.	Saya rasa kecewa	0	1	2	3	4
19.	Saya berputus asa atau tidak bersemangat	0	1	2	3	4
20.	Saya sering terfikir untuk merokok sejak akhir-akhir ini	0	1	2	3	4
21.	Saya sering terasa lapar sejak akhir-akhir ini	0	1	2	3	4
22.*	Saya dapat tidur yang cukup	0	1	2	3	4
23.	Saya sukar memberi tumpuan terhadap sesuatu perkara	0	1	2	3	4
24.*	Saya rasa gembira dan berpuas hati	0	1	2	3	4
25.	Tidur saya sering terganggu sejak akhir-akhir ini	0	1	2	3	4
26.	Saya mempunyai masalah untuk melupakan rokok	0	1	2	3	4
27.	Saya sukar berfikir dengan jelas sejak akhir-akhir ini	0	1	2	3	4
28.	Saya sering terfikir tentang makanan	0	1	2	3	4
